# MT1-MMP as a PET Imaging Biomarker for Pancreas Cancer Management

**DOI:** 10.1155/2018/8382148

**Published:** 2018-08-26

**Authors:** Miguel Ángel Morcillo, Ángel García de Lucas, Marta Oteo, Eduardo Romero, Natalia Magro, Marta Ibáñez, Alfonso Martínez, Guillermo Garaulet, Alicia G. Arroyo, Pedro Pablo López-Casas, Manuel Hidalgo, Francisca Mulero, Jorge Martínez-Torrecuadrada

**Affiliations:** ^1^Biomedical Applications of Radioisotopes and Pharmacokinetics, Research Centre for Energy, Environment and Technology (CIEMAT), Madrid, Spain; ^2^Molecular Imaging Unit, Spanish National Cancer Research Centre (CNIO), Madrid, Spain; ^3^Matrix Metalloproteinases in Angiogenesis and Inflammation Lab, Centro de Investigaciones Biológicas (CIB-CSIC), Madrid, Spain; ^4^Gastrointestinal Cancer Clinical Research Unit, CNIO, Madrid, Spain; ^5^Crystallography and Protein Engineering Unit, CNIO, Madrid, Spain

## Abstract

Pancreatic ductal adenocarcinoma (PDAC) continues to be one of the deadliest cancers for which optimal diagnostic tools are still greatly needed. Identification of PDAC-specific molecular markers would be extremely useful to improve disease diagnosis and follow-up. MT1-MMP has long been involved in pancreatic cancer, especially in tumour invasion and metastasis. In this study, we aim to ascertain the suitability of MT1-MMP as a biomarker for positron emission tomography (PET) imaging. Two probes were assessed and compared for this purpose, an MT1-MMP-specific binding peptide (MT1-AF7p) and a specific antibody (LEM2/15), labelled, respectively, with ^68^Ga and with ^89^Zr. PET imaging with both probes was conducted in patient-derived xenograft (PDX), subcutaneous and orthotopic, PDAC mouse models, and in a cancer cell line (CAPAN-2)-derived xenograft (CDX) model. Both radiolabelled tracers were successful in identifying, by means of PET imaging techniques, tumour tissues expressing MT1-MMP although they did so at different uptake levels. The ^89^Zr-DFO-LEM2/15 probe showed greater specific activity compared to the ^68^Ga-labelled peptide. The mean value of tumour uptake for the ^89^Zr-DFO-LEM2/15 probe (5.67 ± 1.11%ID/g, *n*=28) was 25–30 times higher than that of the ^68^Ga-DOTA-AF7p ones. Tumour/blood ratios (1.13 ± 0.51 and 1.44 ± 0.43 at 5 and 7 days of ^89^Zr-DFO-LEM2/15 after injection) were higher than those estimated for ^68^Ga-DOTA-AF7p probes (of approximately tumour/blood ratio = 0.5 at 90 min after injection). Our findings strongly point out that (i) the *in vivo* detection of MT1-MMP by PET imaging is a promising strategy for PDAC diagnosis and (ii) labelled LEM2/15 antibody is a better candidate than MT1-AF7p for PDAC detection.

## 1. Introduction

Pancreatic adenocarcinoma (PDAC) remains one of the most common and deadly cancers, in great need of optimal diagnostic tools [1]. Abdominal ultrasound is currently the method of choice for an initial examination when pancreatic cancer is suspected, followed by endoscopic ultrasound, contrast-enhanced multidetector computed tomography, or magnetic resonance imaging combined with magnetic resonance cholangiopancreatography for further evaluation once a pancreatic mass is detected [2]. Since these imaging techniques provide only morphological information on the tumour, they are therefore of limited value. Positron emission tomography (PET) could be a valuable alternative imaging technology; however, the metabolic marker most commonly used, 18-fluorodeoxyglucose (^18^F-FDG), lacks sufficient specificity to allow for an accurate diagnosis of PDAC. In addition, the desmoplastic reaction associated with PDAC may cause the malignancy to show up as a hypometabolic lesion under this imaging technique [3]. Additional limitations of ^18^F-FDG include false-positive inflammatory processes and false-negative carcinoma in patients afflicted with diabetes, hyperglycemia, and islet cell tumours. Identification of PDAC-specific molecular markers would then be very useful for improving diagnosis, for identifying those patients more likely to response to certain treatments, and for monitoring therapy progress, which will in turn significantly enhance current survival rates. Because pancreatic cancer is a heterogeneous process of a complex nature, it has not been possible to date to validate any tumour marker as a noninvasive diagnostics tool for this pathology.

Matrix metalloproteinases (MMPs) are a family of zinc-dependent endopeptidases associated with tumour progression and metastasis since they are involved in the degradation of the basement membrane and extracellular matrix (ECM) components. In particular, membrane-tethered MT1-MMP overexpresses in many tumours and associates with tumour growth, invasion, metastasis, and poor prognosis [4, 5]. In PDAC, the observed collagen-mediated upregulation of MT1-MMP in the desmoplastic regions of the tumours promotes both tumour progression and gemcitabine resistance in a PDAC xenograft mouse model [6]. Given that MT1-MMP plays a major role in tumour invasion and metastasis, several pharmaceutical developments have already been carried out using small molecule MT1-MMP inhibitors targeting the active site [7]. This first generation of MMP inhibitors has, however, so far failed to deliver the expected results when assayed in cancer clinical trials due to low net efficacy behaviour and arisen adverse off-target effects since these inhibitors can simultaneously interact with multiple MMPs and other related enzymes harbouring structurally conserved catalytic clefts [8]. This has led to the current exploration of new inhibition strategies based on highly specific antibodies targeting MT1-MMP exosites and allosteric interactions, rather than the catalytic site region, as promising therapeutic alternatives to control MT1-MMP activity [9–12]. Antibody LEM2/15, raised against the exposed surface loop V-B of MT1-MMP [13], has exhibited highly potent and selective inhibition of the metalloproteinase. Protein crystallography studies have accurately detailed the underlying molecular mechanism of inhibition [14], thus supporting the notion that antibody LEM2/15 might be a suitable lead for the development of novel and improved therapeutics.

MT1-MMP would therefore represent a candidate biomarker to facilitate noninvasive PDAC detection, imaging, and the assessment of therapeutic response using specific probes. Several previous studies using MT1-MMP-specific antibodies or peptides have already established the importance of MT1-MMP as an imaging agent in numerous cancer models. Among these, the nonsubstrate peptide termed MT1-AF7p (sequence HWKHLHNTKTFL) that specifically binds to MT-loop of MT1-MMP is worth mentioning. When tagged with a near-infrared fluorescent (NIRF) dye Cy5.5 this peptide provided clear visualization by optical imaging of MT1-MMP-expressing breast tumours in a xenograft mouse model [15]. MT1-AF7p peptide was also used as a radioiodinated peptidic probe for SPECT/CT imaging in a fibrosarcoma mouse model [16] and as a ^99m^Tc-labelled imaging agent for breast cancer diagnosis [17], thus highlighting the potential of using MT1-AF7p for MT1-MMP-targeted tumour detection. This antibody labelling approach has also been explored as probes for the monitoring of MT1-MMP. A ^99m^Tc-labelled anti-MT1-MMP IgG [18] and an ^111^In-labelled miniaturized antibody have been generated for SPECT imaging of fibrosarcoma mouse models where a high accumulation of probes in MT1-MMP-positive areas was observed.

Bearing in mind that PET imaging is far more sensitive and accurate than other imaging techniques in clinic and that it also holds a great promise in the visualization of biology activities, we have developed in this study some MT1-MMP-specific PET probes for pancreatic cancer detection. When designing these types of PET probes, careful consideration should be given beforehand to the values of the half-life of the biomolecule and of the physical life of the positron-emitting radionuclide that are to be linked. Thus, PET radionuclides with longer half-lives, such as ^89^Zr (78.4 hours), are ideally suited for conjugation with intact antibodies (which remain in the blood for long) while smaller peptides (which have shorter clearance times) must be labelled with PET isotopes featuring short or intermediate half-lives such as ^68^Ga. We have recently developed an ^89^Zr-labelled LEM15/2-based probe for the effective monitoring of MT1-MMP by PET in preclinical GBM mouse models [19]. Here, we have tested and assessed this probe in pancreatic cancer mouse models, together with a ^68^Ga-labelled MT1-AF7p peptide. We have compared performance of both probes.

## 2. Materials and Methods

### 2.1. Radiolabelling

#### 2.1.1. Labelling of LEM2/15 Antibody with ^89^Zr

LEM2/15 hybridoma cells were cultured as described [13], and the monoclonal antibodies (mAb) were purified from the hybridoma supernatant following standard methods using protein A-based chromatography. The bifunctional chelator DFO-NCS (Macrocyclics, Dallas, TX) was conjugated to LEM2/15, and subsequent ^89^Zr radiolabelling was performed by adaptation of published protocols [20, 21]. ^89^Zr (*T*_1/2_ = 78.4 h, *β*^+^ = 22.6%; ∼2.7 GBq/ml supplied in 1 M oxalic acid) was obtained from BV Cyclotron VU (Amsterdam, the Netherlands).

For conjugation, 2 mg of LEM2/15 in 1 mL solution at pH 9.0, adjusted with 0.1 M Na_2_CO_3_ (max. 100 *μ*L), were mixed with DFO-NCS (dissolved in DMSO at a concentration 3.5 mM) at a molar ratio of 1 : 5. The reaction was incubated for 40 min at 37°C. Nonconjugated chelator was removed by G25-Sephadex size-exclusion chromatography using a PD-10 column (GE Healthcare Life Sciences) and 5 mg/ml gentisic acid in 0.25 M sodium acetate trihydrate (pH 5.4–5.6) as eluent. For the radiolabelling procedure, the required volume of ^89^Zr-oxalic acid solution corresponding to 37–74 MBq was adjusted to a total volume of 200 *μ*L using 1 M oxalic acid, and 90 *μ*L of 2 M Na_2_CO_3_ were added and incubated for 3 min at room temperature. 1 mL of 0.5 M HEPES and 710 *μ*L of DFO-LEM2/15 (1 mg/mL) were subsequently added and incubated at room temperature for 90 min on a rotating shaker. pH was checked to be at 7.0–7.5. Finally, the reaction mixture was loaded onto a previously equilibrated PD-10 column and eluted with phosphate-buffered saline (PBS) into fractions of 250 *μ*L. After purification, the collected fractions were measured in a dose calibrator (IBC, Veenstra Instruments). Radiochemical purity of the radiolabelled antibody was performed by instant thin-layer chromatography (ITLC) on ITLC strips (model 150–771, Biodex) using 0.02 M citrate buffer (pH 5.0) : acetonitrile (9 : 1) as eluent followed by exposure to phosphor imaging screen-K (Bio-Rad); images were acquired with a Personal Molecular Imager FX system (Bio-Rad).

The stability of ^89^Zr-DFO-LEM2/15 was investigated by incubation in human serum and plasma for 7 days at 4°C and 37°C. The radiochemical purity was determined by ITLC as discussed above.

#### 2.1.2. Labelling of DOTA-AF7p Peptides with ^68^Ga

The peptides DOTA-HWKHLHNTKTFL (denoted as DOTA-AF7p-1) and HWK(DOTA)HLHNTKTFL (denoted as DOTA-AF7p-2) were purchased from Bachem AG (Bubendorf, Switzerland). The molecular mass of both peptides, estimated by MALDI-MS, was 1948.25 and 1948.38 g/mol, respectively; the purity (estimated by HPLC) was 95.0% and 97.7%, respectively, as indicated by the seller.

Radiolabelling of DOTA-AF7p-1 and DOTA-AF7p-2 with ^68^Ga was performed using fractionated elution. The ^68^Ga solution was eluted from a ^68^Ge/^68^Ga-generator, based on nano-SnO_2_ and developed at CIEMAT [22], with 7 mL of 1 M HCl prepared from 30% HCl (JT Baker). Seven vials containing 1 ml eluate each were collected and measured in a dose calibrator. One millilitre of the vial with the highest amount of radioactivity (which contained approximately 85% of total elutable ^68^Ga) was transferred to a reaction vial containing 210 mg (±5 mg) of HEPES (Sigma-Aldrich) dissolved in 0.5 mL deionized water to adjust the pH in the solution at 3–3.5; next, 2–15 nmol of DOTA-peptide dissolved in deionized water (0.1 nmol/*µ*L) was added to the radiolabelling reaction mixture. After very careful shaking, the complex formation was performed at 90 ± 5°C for 5 min using a microwave with monomodal radiation and then cooled to room temperature with nitrogen.

The labelled peptides were purified using a Sep-Pak Light C18 cartridge (Waters) conditioned and equilibrated previously with 4 mL of pure ethanol and 4 mL of deionized water. Thus, the reaction mixture was transferred onto Sep-Pak where the ^68^Ga-DOTA-AF7p and colloidal ^68^Ga were retained, whereas free ^68^GaCl_3_ passed through the cartridge [23]. Next, the cartridge was rinsed with 4 mL of deionized water, and the activity on the Sep-Pak cartridge was recovered with 0.5 mL ethanol 96% (v/v). Finally, ethanol was evaporated to dryness, and ^68^Ga-DOTA-AF7p was dissolved in 0.9% saline solution.

Radiochemical yield was determined as % activity recovered in ethanol with respect to total activity used, after the radioactivity was decay-corrected to the start of procedure. An aliquot was taken to analyse the radiochemical purity (RQP) of the final product by radio-HPLC. The radio-HPLC method used a Jasco HPLC system equipped with a photodiode array UV-detector MD-4015 (Jasco), a radioactivity detector LB 507A (Berthold), and a reversed-phase Phenomenex Jupiter Proteo 90 Å column (4 *µ*m, 250 × 4.6 mm). The flow rate was 1 mL/min. The mobile phase was 95% solvent A (0.1% TFA in 5% acetonitrile) and 5% solvent B (0.1% TFA in 95% acetonitrile). The linear gradient started 2 min after sample injection with 95% solvent A and 5% solvent B to 0% solvent A and 100% solvent B at 12 min, maintained at 15 min and followed with 95% solvent A and 5% solvent B at 18 min.

### 2.2. Cell Culture

Human pancreas adenocarcinoma cell line Capan-2 was obtained from the American Type Culture Collection (ATCC HTB-80™) and cultured at 37°C in 5% CO_2_ humidity according to standard mammalian tissue culture protocols in McCoy's 5A medium (Gibco) supplemented with 20% fetal bovine serum (FBS) (Gibco), 100 units/ml penicillin/100 *µ*g/ml streptomycin (Sigma), 2 mM L-glutamine (Gibco), and 10 mM Hepes buffer (Sigma).

### 2.3. Tumour-Bearing Mice (CAPAN-2 and Patient-Derived Xenografts)

All animal experimental procedures were performed by the following protocols approved by the CNIO-ISCIII Ethics Committee for Research and Animal Welfare (CEIyBA) and the CIEMAT Animal Ethical Committee; they were performed in strict adherence to the guidelines stated in the International Guiding Principles for Biomedical Research Involving Animals, established by the Council for International Organizations of Medical Sciences (CIOMS). All animal experimental procedures had also been approved by the Competent Authority of the Regional Government of Madrid (CAM), Spain (projects PROEX 104/16 and PROEX 094/15).

Three tumour models were used: (i) CAPAN-2 subcutaneous, (ii) subcutaneous patient-derived xenograft (PDX), and (iii) orthotopic PDX models.

For the cell line-derived model, 1 × 10^6^ CAPAN-2 cells were injected subcutaneously into the flanks of female athymic nude-*Foxn1nu* (*nu/nu*) mice (Harlan Laboratories). Tumours were allowed to develop until palpable prior to PET imaging. Mice were sacrificed when the tumour mass reached a maximum size of 1500 mm^3^ or tumour ulceration was observed, or mice were symptomatic from their tumours which included signs of lethargy, poor grooming, weight loss, and hunching.

PDX models used in this work were established as described by Rubio-Viqueira et al. [24] and Hidalgo et al. [25] using four- to six-week-old female athymic nude-*Foxn1nu* (*nu/nu*) mice, purchased from Envigo (Barcelona, Spain). Animals were maintained at the Spanish National Cancer Research Centre (CNIO) Animal Facility (awarded with the AAALAC accreditation). Orthotopic tumours were monitored by ultrasonography, and those implanted into the flanks were allowed to grow to approximately 40 to 200 mm^3^ prior to perform PET imaging studies.

### 2.4. PET/CT Imaging

PET imaging was performed with a small-animal Argus PET-CT scanner (SEDECAL, Madrid, Spain). PET studies (energy window 250–700 KeV and 30 min static acquisition) and CT studies (voltage 45 kV, current 150 *μ*A, 8 shots, 360 projections, and standard resolution) were performed either at 90 min or at various time points after injection of ^68^Ga-DOTA-AF7p or ^89^Zr-DFO-LEM2/15, respectively, in mice anesthetized by inhalation of 2–2.5% isoflurane. PET image reconstruction was accomplished using a 2D-OSEM (ordered subset expectation maximization) algorithm (16 subsets and two iterations), with random and scatter correction. A calibration factor predetermined by scanning a cylindrical phantom containing a known activity of ^68^Ga or ^89^Zr was used to convert counts per pixel/sec to kBq/cm^3^. Manually drawn regions of interest (ROIs) selected from PET images using CT anatomical guidelines were used to determine the mean radiotracer accumulation in units of %ID/g tissue (decay corrected to the time of injection) by dividing the obtained average tracer concentration (kBq/cm^3^) in the region by the total ID (injected dose, expressed in kBq). Tumour-to-blood and tumour-to-background ratios were calculated from %ID/g values in ROIs for the whole tumour, heart (a measure of the blood pool), and brain (for background measurements). Images were analysed with the image analysis software ITK-SNAP [26] (http://www.itksnap.org).

### 2.5. Immunohistochemistry

Tumour-bearing mice were sacrificed after acquisition of the last PET image, and tumours were excised, fixed in 10% buffered formalin (Sigma), and embedded in paraffin. For MT1-MMP expression analysis, we performed immunohistochemical staining with anti-MT1-MMP LEM2/15 antibody at 1 : 400 dilution after antigen retrieval with low pH buffer in Autostainer platform (Dako) and counterstaining with hematoxylin. Slides were digitalized in the Mirax Scan (Carl Zeiss AG, Oberkochen, Germany), and pictures were registered with the Pannoramic Viewer software (3DHistech Ltd., Ramsey, NJ, USA).

### 2.6. Statistics

Univariate methods such as the *t*-test and ANOVA were used to compare means of one variable across two or more groups. The significance of differences was tested at the 95% confidence level by Bonferroni's multiple comparisons test; a *P* value of 0.05 or less was considered significant. Data in graphs are presented as the mean ± SD. The Grubbs test was applied to detect eventual outliers in data sets with the online “Outlier Calculator” tool provided by GraphPad software (http://www.graphpad.com/quickcalcs/Grubbs1.cfm).

## 3. Results and Discussion

Initial studies on ^89^Zr-labelled LEM2/15, an antihuman MT1-MMP mAb, as an immunoPET probe for glioblastoma in xenograft mice were previously reported by our group [19]. Now in this study, the use of ^89^Zr-DFO-LEM2/15 was validated as an immunoPET probe in CAPAN-2 cell line as well as in patient-derived pancreatic ductal adenocarcinoma xenografts. The *in vivo* PET imaging of ^89^Zr-DFO-LEM2/15 was additionally compared with that of ^68^Ga-DOTA-AF7p, a peptide that binds to the MT-loop region of MT1-MMP [15].

### 3.1. Probe Radiosynthesis

#### 3.1.1. ^89^Zr-DFO-LEM2/15

**Figure 1:**
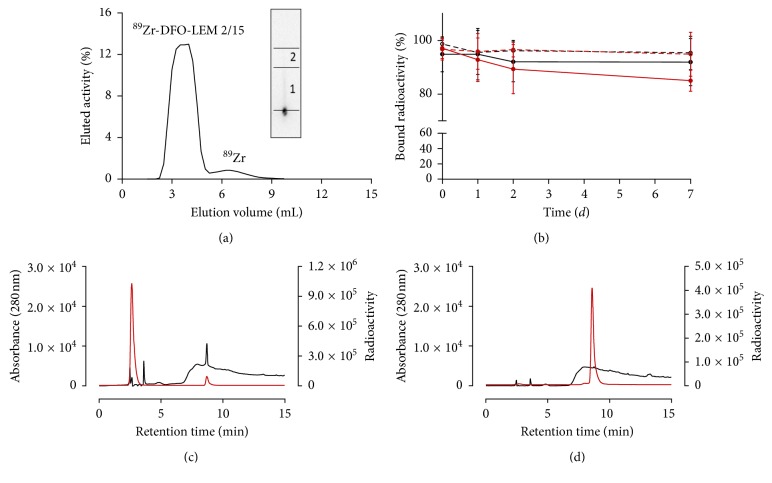
(a) Purification of ^89^Zr-DFO-LEM2/15 in a PD-10 column; the antibody elutes between volume fractions 2.5 and 5.0 ml. Inset: ITLC image illustrating RQP of the ^89^Zr-DFO-LEM2/15 peak. 1 corresponds to the radioimmunoconjugate and 2 to the free radionuclide. (b) Percentage of bound radioactivity to LEM2/15 after 7-day incubation of ^89^Zr-DFO-LEM2/15 with human serum at 37°C (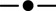) and 4°C (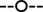) and human plasma at 37°C (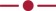) and 4°C (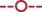); data expressed as mean ± SD. (*n*=4). Representative HPLC chromatogram of ^68^Ga-DOTA-AF7p-1 before (c) or after (d) to Sep-Pak Light C18 cartridge purification. ^68^Ga-DOTA-AF7p-1 is detected at 8.7 min by UV absorbance at 280 nm (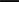) and radioactivity detector (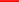).

Conjugation of LEM2/15 to desferrioxamine was achieved via an N-succinimidyl linkage in an mAb : deferroxamine ratio of approximately 1 : 2-3 as reported previously [19]. ^89^Zr-DFO-LEM2/15 was successfully synthesized at a radiochemical yield of >85%. After PD-10 column purification, RQP was determined by ITLC to be above 95% (Figure 1(a)). The specific activity of final product was measured to be 9.675 ± 2.445 MBq/nmol (*n*=3).

The stability of the radiolabelled antibody was assessed with ITLC.  Figure 1(b) illustrates that during the 7-day incubation period at 37°C, the ^89^Zr-DFO-LEM2/15 in human serum and plasma decreased slightly from 94.9 ± 6.5% and 97.1 ± 3.9% at the beginning, respectively, to values of 91.9 ± 8.8% and 85.0 ± 3.9%, respectively. Similarly, the amount in human serum and plasma decreased from 98.7 ± 1.5% and 96.7 ± 4.0% at the beginning, respectively, to 95.4 ± 6.2% and 94.9 ± 8.2%, respectively, during the 7-day incubation period at 4°C. Therefore, the data indicated that probe displayed a high kinetic stability and was suitable for further *in vivo* studies.

#### 3.1.2. ^68^Ga-DOTA-AF7p

The radiochemical yield of both ^68^Ga-labelled DOTA-peptides, DOTA-HWKHLHNTKTFL (DOTA-AF7p-1) and HWK(DOTA)HLHNTKTFL (DOTA-AF7p-2), was 30.1 ± 17.9% (*n*=29) indicating a high interexperiment variability in the radiolabelling procedures. No statistically significant differences between the two peptides were found. The radiochromatogram of the reaction mixture prior to passing through the Sep-Pak Light C18 cartridge showed two distinct peaks corresponding to free ^68^Ga and ^68^Ga-DOTA-AF7p-1 with retention times of 2.5 and 8.7 min, respectively.  Figure 1(c) shows a representative chromatogram of peptide labelling with a low yield aiming at demonstrating that even in those cases, a final product can be successfully obtained. After purification, the radiochromatogram showed just one single peak corresponding to the radiolabelled probe (Figure 1(d)); RQP was greater than 95%. The radiochromatogram corresponding to ^68^Ga-DOTA-AF7p-2 is not presented because it was similar to that of ^68^Ga-DOTA-AF7p-1. The specific activity of the final product once radiolabelling was completed was estimated to be 8.4 ± 6.0 MBq/nmol (*n*=29).

### 3.2. ^68^Ga-DOTA-Peptides PET Imaging

#### 3.2.1. CAPAN-2-Tumour-Bearing Mice

**Figure 2:**
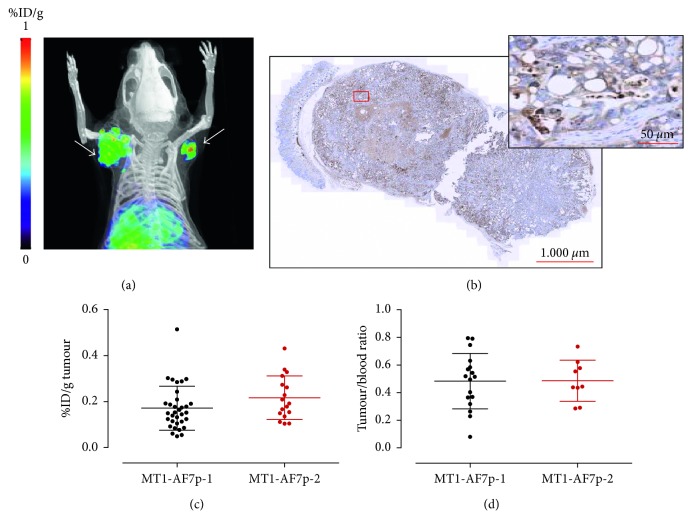
CAPAN-2-tumour-bearing mice injected with ^68^Ga-DOTA-AF7p probes. (a) PET/CT image of representative mouse with subcutaneous CAPAN-2 xenograft (white arrows) that was injected with ^68^Ga-DOTA-AF7p-1. Image was acquired 90 min after injection. (b) Immunohistochemistry of tumour tissue from xenografted mice. MT1-MMP was detected using LEM2/15 antibody. Scale bar: 1000 *µ*m. Higher magnification of the boxed area is shown at the right corner. Scale bar: 50 *µ*m. (c) Tumour uptake (expressed as %ID/g) of ^68^Ga-DOTA-AF7p-1 and ^68^Ga-DOTA-AF7p-2 probes as quantified by PET imaging. (d) Tumour-to-blood ratio derived from PET images for both radiolabelled probes.

Most of the current understanding of cancer and its hallmarks is based on the establishment of long-term *in vitro*-cultured tumour cell lines and their *in vivo* inoculation in mice. Ellenrieder et al. [27] reported high levels of MT1-MMP expression in most pancreatic cancer cell lines including CAPAN-2. To evaluate if both radiolabelled peptides could image MT1-MMP expression *in vivo* by PET, we administered 4 ± 3 MBq (*n*=25) of ^68^Ga-DOTA-AF7p-1 or ^68^Ga-DOTA-AF7p-2 intravenously into mice bearing CAPAN-2 bilateral flank xenografts (*n*=9), and we performed PET/CT imaging at distinct time points for a month. *In vivo* small-animal PET imaging showed accumulation of radiolabelled peptide in CAPAN-2 tumours, and we did not find differences in the tumour uptake visualization between the two radiolabelled peptides.  Figure 2(a) shows a representative example.

The tumour uptake of ^68^Ga-DOTA-AF7p-1 and ^68^Ga-DOTA-AF7p-2 was found to be very similar, and there were no statistically significant differences between the two radiolabelled peptides, 0.171 ± 0.095 (*n*=33) and 0.216 ± 0.095 (*n*=17) %ID/g for ^68^Ga-DOTA-AF7p-1 and ^68^Ga-DOTA-AF7p-2, respectively (Figure 2(c)). This is in good agreement with the results obtained by Kondo et al. [16], who developed a radiolabelled peptide probe by adding a Cys residue at the N-terminus of the peptide MT1-AF7p to allow facile radiolabelling of the thiol moiety by *N-*(*m-*[^123/125^I]iodophenyl) maleimide ([^123/125^I]IPM) for SPECT/CT imaging in HT1080-tumour-bearing mice (HT1080 is a human fibrosarcoma cell line known to highly express MT1-MMP); the radiolabelled probe was therefore similar to DOTA-AF7p but with IPM instead of DOTA. Kondo et al. [16] determined that the tumour accumulation at 60 and 120 min after injection was of 0.19 ± 0.06 and 0.10 ± 0.01%ID/g, respectively. These findings, however, clash with the results reported by Min et al. [17] which used HYNIC as the bifunctional chelating agent (instead of DOTA or IPM) and tricine/TPPTS as coligands to prepare [^99m^Tc]-(HYNIC-AF7p)(tricine)(TPPTS) for *in vivo* SPECT imaging of breast cancer. They found a highly tumour uptake of the probe (2.18 ± 0.11%ID/g at 2 h post-injection) in MDA-MB-231-tumour-bearing mice. These differences in the uptake of the AF7p tracer can be due to many parameters playing a role in targeting such as the diverse density of MT1-MMP in the tissues, the affinity of peptide after labelling, the rate of tracer delivery, the vascular permeability of tumours, and the interstitial pressure among others [28].

As for the tumour-to-blood ratio, often used as a measure of image contrast, we did not observe any statistically significant differences between ^68^Ga-DOTA-AF7p-1 and ^68^Ga-DOTA-AF7p-2 (0.48 ± 0.20 and 0.49 ± 0.15, resp.) at 90 min after injection (Figure 2(d)). This ratio was similar to that found by Kondo et al. [16] and lower than that determined by Min et al. [17].

All tumours contained tumour cells embedded in desmoplastic tumour stroma, a typical feature of pancreatic tissue. Immunohistochemical examination of tumour sections for MT1-MMP revealed expression of MT1-MMP in tumour cells but not in stromal cells (Figure 2(b)).

#### 3.2.2. Heterotopic Patient-Derived Xenograft Model

**Figure 3:**
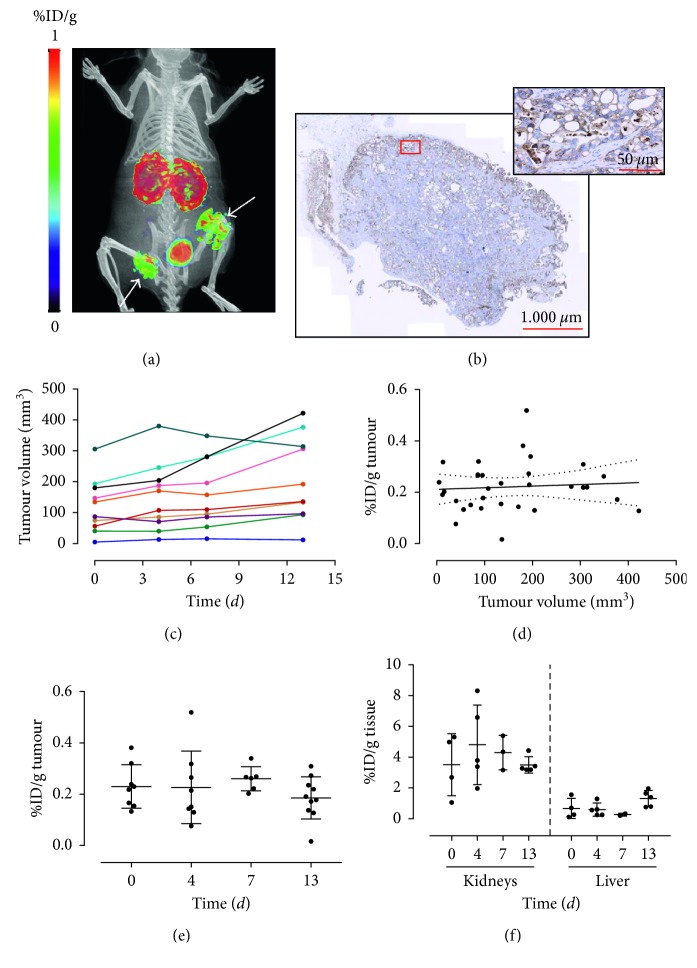
Heterotopic patient-derived xenografted mice injected with ^68^Ga-DOTA-AF7p-1. (a) PET/CT image of representative heterotopic PDX mouse injected with ^68^Ga-DOTA-AF7p-1 acquired 90 min after injection. Tumour locations are indicated by white arrows. (b) Immunohistochemistry of tumour tissue from PDX mice. MT1-MMP was detected using LEM2/15 antibody. Scale bar: 1000 *µ*m. Higher magnification of the boxed area is shown at the right corner. Scale bar: 50 *µ*m. (c) Tumour volume was measured by CT four times after onset of PET imaging study. Each tumour is represented by a different colour line on the graph. (d) Tumour uptake (quantified by PET imaging and expressed as %ID/g) as a function of tumour volume for all scanned mice, showing that there is no relationship between tumour size and ^68^Ga-DOTA-AF7p-1 uptake. (e) Variation of tumour uptake of ^68^Ga-DOTA-AF7p-1 along the PET imaging study. (f) Variation of kidneys and liver uptakes of ^68^Ga-DOTA-AF7p-1 along the PET imaging study.

Patient-derived xenografts (PDXs) are typically generated by the subcutaneous implantation of fresh, surgically derived human tumour material into immunodeficient mice. PDXs retain stably molecular, genetic, and histopathological features of their originating tumours and therefore are currently the only model system able to incorporate directly the vast interpatient and intratumour heterogeneity inherent to human cancer [29]. With the aim to determine the applicability of PET imaging of PDAC tumours in a more clinically relevant model with ^68^Ga-DOTA-AF7p, we performed PET/CT imaging with this radiolabelled probe at four-time points during two weeks in patient-derived tumour-bearing mice (*n*=5). As we did not find any statistically significant differences between the two radiolabelled peptides tested in CAPAN-2-bearing tumour mice, we decided to carry out our PET/CT studies after injecting 1.9 ± 1.8 MBq (*n*=17) of ^68^Ga-DOTA-AF7p-1.  Figure 3(a) shows a representative PET/CT image taken 90 min after injection. After performing *in vivo* imaging, we also assessed the overexpression of MT1-MMP in patient-derived tumours by means of immunohistochemistry making use of the LEM2/15 antibody. Immunohistochemistry analyses revealed that MT1-MMP localized to the membranes of pancreatic ductal cells while no expression was detected in the stromal cells (Figure 3(b)).

Each mouse carried two patient-derived tumours implanted bilaterally subcutaneously. We used CT to evaluate volume growth; Jensen et al. had demonstrated that microCT was a more accurate than other methods (as external calliper) for the *in vivo* volumetric measurements of subcutaneous tumours in mice [30]. All mice developed subcutaneous tumours in both flanks, with a mean tumour volume that increased from 122 ± 89 mm^3^ to 208 ± 137 mm^3^ along the whole duration of the PET imaging study (Figure 3(c)). Although a variable tumour volume between repeated scans may *per se* cause variability in the tumour uptake of ^68^Ga-DOTA-AF7p-1, our analysis revealed the absence of significant correlation between the tumour uptake and tumour size (Figure 3(d)). At the start of the PET study, the tumour displayed an uptake of 0.230 ± 0.085%ID/g (*n*=8) and it remained at this level throughout the time course: 0.227 ± 0.142 (*n*=8), 0.260 ± 0.047 (*n*=6), and 0.185 ± 0.082 (*n*=10) %ID/g tumour at 5, 8, and 14 days, respectively (Figure 3(e)). The mean tumour uptake of ^68^Ga-DOTA-AF7p-1 was of 0.221 ± 0.096%ID/g tumour (*n*=32). This is in good agreement with the results reported by Kondo et al. [16] in their study of CAPAN-2-bearing tumour xenograft mice. The mean value of the tumour-to-blood ratio at 90 min ^68^Ga-DOTA-AF7p-1 after injection in PDX (1.36 ± 0.98, *n*=26) was higher than that measured in CAPAN-2-bearing tumour mice (0.48 ± 0.20, *n*=17), demonstrating that for the PET visualization of the radiolabelled probe, the PDX model is better one than the CAPAN-2-derived model.

It should be noted that in addition to the tumour, kidneys and bladder also showed signals, indicating that the tracer is mainly excreted through the renal route (Figure 3(f)). The mean accumulation of the probe was significantly lower in liver than in kidneys (0.80 ± 0.60, *n*=16 versus 4.03 ± 1.73, *n*=17 resp.).

We are aware of, at least, one limitation in our research, that is the relatively low tumour accumulation level of both radiolabelled peptides, which was also occurred in the study of Kondo et al. [16]. A setup allowing for an increase of the ^68^Ga-DOTA-AF7p uptake in pancreatic tumours would be required if we were to carry out further studies. A possible strategy could be to increase the affinity of the radiolabelled probe for MT1-MMP inserting a spacer linker that imposes a separation between the bifunctional chelating agent (DOTA) and the peptide, as it has already been suggested by several other authors [16, 31]. Our study is nevertheless the first report of the use of a radiolabelled peptide probe for the PET imaging of MT1-MMP in PDAC cell- and patient-derived xenograft mice.

### 3.3. ^89^Zr-DFO-LEM2/15 PET Imaging

#### 3.3.1. CAPAN-2-Tumour-Bearing Mice

**Figure 4:**
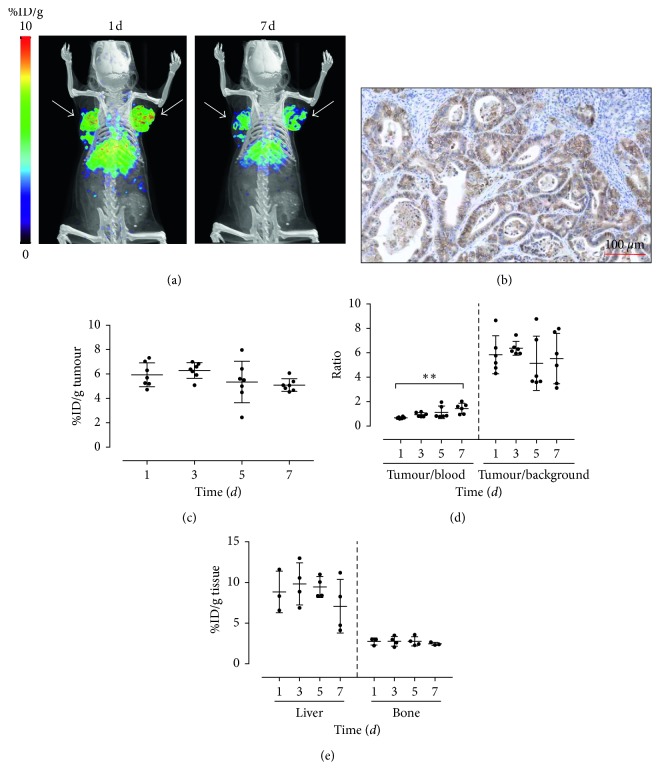
CAPAN-2-tumour-bearing mice injected with ^89^Zr-DFO-LEM2/15. (a) Representative PET/CT images of a mouse with subcutaneous CAPAN-2 xenograft injected with ^89^Zr-DFO-LEM2/15 acquired at 1 and 7 days after injection. Tumour locations are indicated by white arrows. (b) Immunohistochemistry of tumour tissue from xenografted mice. MT1-MMP was detected using LEM2/15 antibody. Scale bar: 100 *µ*m. (c) Tumour uptake (expressed as %ID/g) of ^89^Zr-DFO-LEM2/15 as quantified by PET imaging. (d) Tumour-to-blood and tumour-to-background ratios derived from PET images. (e) Liver and bone uptake of ^89^Zr-DFO-LEM2/15 as quantified by PET imaging at different times after injection.

We had already reported the quantitative assessment of MT1-MMP expression by means of immunoPET using a specific mAb (LEM 2/15), readily labelled with ^89^Zr and employed in the *in vivo* preclinical imaging of glioblastoma MT1-MMP^+^ tumours [19]. ^89^Zr-DFO-LEM 2/15 small-animal PET was conducted on mice harbouring xenografted tumours at opposite flanks with CAPAN-2 cells (*n*=4) after injection of 0.55 ± 0.01 MBq (*n*=4).  Figure 4(a) presents some typical images of a CAPAN-2-tumour-bearing nude mouse at 1 and 7 days after injection, showing an excellent visualization of tumours. Levels of radioactivity in tumours, tumour-to-blood, and tumour-to-background ratios were estimated from PET images. In tumours, radioactivity did not vary significantly in a time-dependent manner, with the uptake being 5.93 ± 0.98, 6.29 ± 0.64, 5.35 ± 1.70, and 5.09 ± 0.52%ID/g (*n*=7) at 1, 3, 5, and 7 days after injection, respectively, in CAPAN-2 tumours (Figure 4(c)). The mean value of tumour uptake for the ^89^Zr-DFO-LEM 2/15 probe (5.67 ± 1.11%ID/g, *n*=28) was 25–30 times higher than that for the ^68^Ga-DOTA-AF7p ones. Sections of tumours immunostained for MT1-MMP confirmed the expression of this protein in the tumour cells but not in the stromal cells (Figure 4(b)) as we have remarked earlier on in this report (Figure 2(b)).

Tumour/blood ratios in CAPAN-2 tumours increased lightly with time (0.67 ± 0.08, 0.93 ± 0.17, 1.13 ± 0.51, and 1.44 ± 0.43 at 1, 3, 5, and 7 d ^89^Zr-DFO-LEM 2/15 after injection, resp.) (Figure 4(d)). These values also were higher than those estimated for ^68^Ga-DOTA-AF7p probes (approximately tumour/blood ratio = 0.5 at 90 min after injection). These results indicate that the visualization and quantification of the tumour uptake by noninvasive PET imaging is better with ^89^Zr-DFO-LEM 2/15 than it is with ^68^Ga-DOTA-AF7p. The difference in signal between tumour and normal tissue in the context of nuclear medicine imaging depends upon the tumour-localizing agent. Tumour-to-background ratios can be anything from 1 : 1 to >10 : 1 when using radiolabelled antibodies [32]. Thus, tumour/background ratios in CAPAN-2 tumours exhibited high values (5.85 ± 1.56, 6.37 ± 0.57, 5.14 ± 2.23, and 5.53 ± 2.06 at 1, 3, 5, and 7 days after injection, resp.) (Figure 4(d)) confirming previous findings.

In addition to the tumour uptake, visible signal was observed in the liver due to the metabolization and the nonspecific clearance of the radiolabelled antibody, which is a characteristic feature of the distribution pattern of monoclonal antibodies (Figure 4(e)). Liver uptake of ^89^Zr-DFO-LEM 2/15 at 1, 3, 5, and 7 days after injection was 8.84 ± 2.56, 9.83 ± 2.59, 9.46 ± 1.30, and 7.08 ± 3.30%ID/g, respectively. Lastly, bone uptake was low and did not increase over time, in support of the notion of the *in vivo* stability of the radioimmunoconjugate (free Zr^4+^ salts can manifest tropism towards the bone); the ^89^Zr-DFO-LEM 2/15 uptake was 2.75 ± 0.44, 2.78 ± 0.61, 2.77 ± 0.58, and 2.46 ± 0.19 at 1, 3, 5, and 7 days after injection, respectively (Figure 4(e)).

^89^Zr immunoPET continues to drive research and development in the field of the application of mAbs in PET molecular imaging [33] and has shown great potential in cancer imaging [34]. Although ^89^Zr-immunoPET is a highly attractive technique to measure tumour-associated antigens as well as the *in vivo* distribution of mAbs, it has to be mentioned that it is restrained by a few limitations. First of all, the enhanced permeability and retention effect characteristic of the leaky nature of tumour vasculature and reduced lymphatic drainage [35] may result in nonreceptor-mediated uptake of radiolabelled antibodies producing, thus, false-positive results. Second, a low target expression may lead to false-negative results due to low imaging contrast derived from the long circulatory half-life of monoclonal antibodies [32]. Finally, as a longer-lived medical radionuclide, one of the principal concerns regarding the use of ^89^Zr in the clinic is the substantial radiation dose that patients receive in comparison to shorter-lived nuclides. However, as long as the radiotracer clears the healthy tissue as expected, the prevailing viewpoint is that the increased diagnostic value is worth the additional dose [36]. Along these lines, the uptake in bones is a notable issue with ^89^Zr PET imaging in preclinical mouse models. Biodistribution analyses commonly show up to 10% injected dose per gram activity in the bone [34], which is consistent with the results we have obtained in our research. Localization of radioactivity to the bone is disadvantageous since it implies that an increased radioactivity dose is delivered to the bone marrow. To overcome such limitations, our current work aims at extending these initial findings using engineered and miniaturized derivatives of the LEM2/15 antibody. The miniaturized antibodies could improve the tumour penetration ratio and therefore result in an improved tumour-targeted imaging [37]. Moreover, labelling with PET isotopes characterized by short or intermediate half-lives such as ^68^Ga would optimize the pharmacokinetics and facilitate wide access to this technology since ^68^Ga can be easily produced at most facilities using a ^68^Ge/^68^Ga generator system, thus avoiding the complexity of a cyclotron facility. They facilitate the implementation of a same-day imaging approach, much like the current practice for ^18^F-FDG-PET, and their employment leads to a lower radiation exposure versus immunoPET with intact antibodies, making them appealing for routine use in the clinic [38]. This approach is very promising to assess target expression levels in individual patients so to identify patients that will likely benefit from targeted treatments.

#### 3.3.2. Orthotopic Patient-Derived Xenograft Model

**Figure 5:**
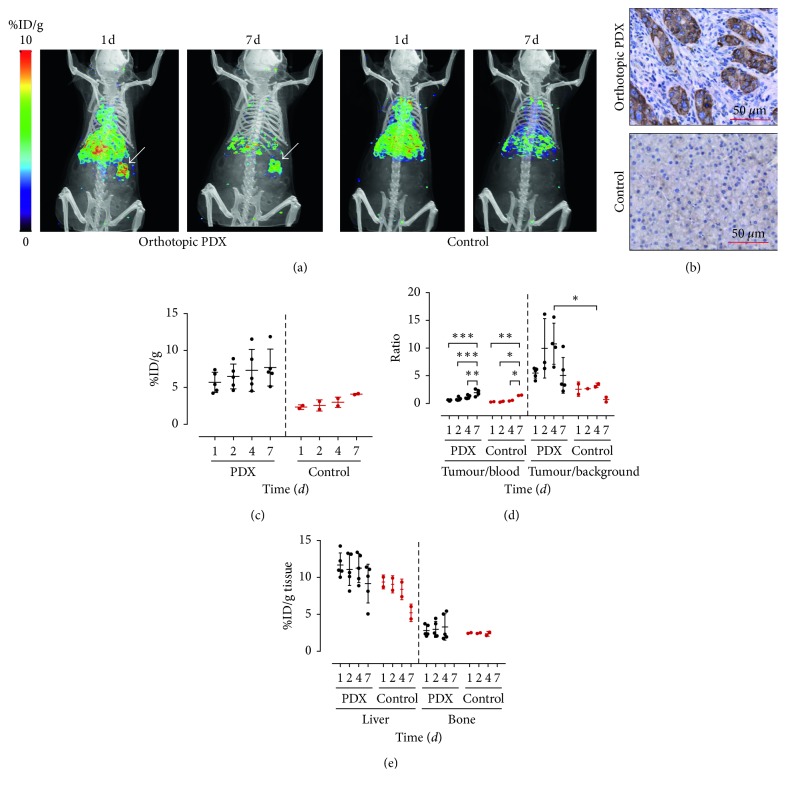
Orthotopic patient-derived xenografted mice injected with ^89^Zr-DFO-LEM2/15. (a) Representative PET/CT images of orthotopic PDX and control mice injected with ^89^Zr-DFO-LEM2/15 acquired at 1 and 7 days after injection. Tumour locations are indicated by white arrows. (b) Immunohistochemistry studies of pancreas tissues derived from orthotopic PDX and control mice. MT1-MMP was detected using LEM2/15 antibody. Scale bar: 50 *µ*m. (c) Tumour uptake (expressed as %ID/g) of ^89^Zr-DFO-LEM2/15 as quantified by PET imaging. (d) Tumour-to-blood and tumour-to-background ratios derived from PET images. (e) Liver and bone uptake of ^89^Zr-DFO-LEM2/15 as quantified by PET imaging at different times after injection in PDX and control mice.

To evaluate the ability of ^89^Zr-DFO-LEM2/15 to noninvasively determine MT1-MMP protein levels in nude mice bearing orthotopic PDX tumours (*n*=5) as described above, the radioimmunoconjugate was prepared and injected (1.8 ± 1.6 MBq, *n*=7) to the subject mice once the presence of tumours was confirmed by ultrasonography, and serial PET imaging was performed at 1, 2, 4, and 7 days. ^89^Zr-DFO-LEM2/15 was also injected in mice free of tumours (*n*=2) used as a control group. The PET images presented in Figure 5(a) were taken at different time points after injection and are compared to control mice; they show that the tumours were clearly visualized in orthotopic PDX mice after injection of ^89^Zr-labelled mAb LEM2/15. Tumour uptake increased over time, from 5.7 ± 1.4%ID/g at 1 d to 7.7 ± 2.5%ID/g at 7 d in PDX mice, whereas the accumulation in pancreas of control mice increased from 2.3 ± 0.3%ID/g at 1 d to 4.1 ± 0.1%ID/g at 7 d (Figure 5(c)); thus, ^89^Zr-DFO-LEM2/15 uptake in tumours of PDX mice was about twice higher than in the pancreas of control mice. The tumour-to-blood ratios were at all time points higher in PDX mice than in control ones, 0.56 ± 0.10, 0.81 ± 0.28, 1.15 ± 0.32, and 1.95 ± 0.63 versus 0.28 ± 0.05, 0.31 ± 0.13, 0.51 ± 0.09, and 1.47 ± 0.05 at 1, 2, 4, and 7 days, respectively (Figure 5(d)). Tumour-to-background ratios were also around 2–7 times higher in PDX mice than in control mice (Figure 5(d)). The mean values of radioimmunoconjugate tumour uptake, tumour-to-blood, and tumour-to-background ratios in PDX mice were similar to those found in CAPAN-2-tumour-bearing mice. As for the liver and bone uptake of ^89^Zr-DFO-LEM2/15 in mice bearing orthotopic PDX tumours (Figure 5(e)), the mean values were also similar to those shown in Figure 5(e).

We also assessed by immunochemistry techniques the expression of MT1-MMP in tumours of PDAC patient-derived xenograft mice (Figure 5(b)). As expected, whereas MT1-MMP was highly expressed in tumour cells but not in stromal cells, verified by immunostaining using LEM2/15, no immunostaining was observed in the pancreas of control mice. This is in good agreement with studies showing that MT1-MMP is overexpressed in pancreatic tumours relative to normal pancreas [39].

Our preclinical studies with MT1-MMP overexpressing CAPAN-2 cells and PDAC patient-derived tumours demonstrate that ^89^Zr-DFO-LEM2/15 constitutes a promising radiotracer for noninvasive immunoPET measurements of MT1-MMP expression *in vivo*. Given the crucial role that MT1-MMP plays in both physiologic and pathologic conditions, MT1-MMP overexpression is seen in a number of different tumours, including PDACs as shown herein and in earlier reports [39–41]. Pancreatic ductal adenocarcinoma is the most common form of pancreatic cancer and accounts for ∼90% of all pancreatic tumours [42]. It is associated with an overall 5-year survival rate of <8%, exhibiting the poorest prognosis of all solid tumours [43]. One of the reasons for this poor prognosis is the high resistance of PDAC to conventional chemotherapy treatments [44]. Following the initial success of gemcitabine in the treatment of advanced PDAC, combination therapies with gemcitabine were administered with limited success to tackle locally advanced and metastatic disease. This failure is attributable to many factors, including extrinsic or intrinsic resistance to gemcitabine [45]. Notably, PDAC is a tumour characterized by the development of extensive fibrosis termed desmoplasia. Interestingly, MT1-MMP overexpression was particularly prominent in areas of the tumour with intense fibrotic reaction, suggesting that type I collagen-enriched desmoplasic reaction may also contribute to MT1-MMP expression in the *in vivo* setting [40]. Dangi-Garimella et al. [6] showed that MT1-MMP expression associates to an increased HMGA2 expression (a nonhistone DNA-binding nuclear protein involved in chromatin remodelling and gene transcription) in human PDAC tumours, suggesting that the marked fibrotic reaction may contribute to gemcitabine resistance through increased MT1-MMP-HMGA2 signalling. Therefore, owing to the central role that MT1-MMP plays in collagen-induced gemcitabine resistance [45], this metalloproteinase emerges as a good predictive biomarker of the response to gemcitabine in patients with pancreatic cancer; thus, PET imaging probes such as ^68^Ga-DOTA-AF7p or ^89^Zr-DFO-LEM2/15, with the modifications that our research has highlighted so as to increase the affinity of ^68^Ga-DOTA-AF7p and/or obtain miniaturized derivatives of the LEM2/15 mAb, could be used for the early prediction of resistance to gemcitabine in PDAC patients. Targeting MT1-MMP with these probes could furthermore also be a novel approach to sensitizing pancreatic tumours to gemcitabine.

## 4. Conclusions

We have in this report radiolabelled two molecules, an anti-MT1-MMP monoclonal antibody (LEM2/15) and a MT1-MMP-specific binding peptide (MT1-AF7p) with ^89^Zr and ^68^Ga, respectively. We assessed and compared their *in vivo* properties for use as new MT1-MMP-targeted PET imaging probes in several models of pancreatic cancer. Both tracers highly accumulated in MT1-MMP-expressing tumours and were able to visualise clearly subcutaneously and orthotopically implanted xenografts, although the ^89^Zr-DFO-LEM2/15 probe exhibited much greater specific uptake compared to the ^68^Ga-labelled peptide. The results of our study highlight the relevance of MT1-MMP as a suitable biomarker for noninvasive PET imaging of pancreatic cancer. Also, our results suggest that the ^89^Zr-DFO-LEM2/15 probe may have potential application in PDAC detection and follow-up and can be a lead for further tracer optimization. In fact, miniaturization of LEM2/15 and radiolabelling with ^68^Ga to enhance its radiopharmacological properties are in progress and may facilitate translation in the clinical setting for the improvement of the clinical management of PDAC patients.

## Data Availability

The data used to support the findings of this study are available from the corresponding author upon request.

## References

[1] Ryan D. P., Hong T. S., Bardeesy N. (2014). Pancreatic adenocarcinoma. *New England Journal of Medicine*.

[2] Chu L. C., Goggins M. G., Fishman E. K. (2017). Diagnosis and detection of pancreatic cancer. *Cancer Journal*.

[3] Sahani D. V., Bonaffini P. A., Catalano O. A. (2012). State-of-the-art PET/CT of the pancreas: current role and emerging indications. *RadioGraphics*.

[4] Mimori K., Fukagawa T., Kosaka Y. (2008). A large-study of MT1-MMP as a marker for isolated tumor cells in peripheral blood and bone marrow in gastric cancer cases. *Annals of Surgical Oncology*.

[5] Perentes J. Y., Kirkpatrick N. D., Nagano S. (2011). Cancer cell-associated MT1-MMP promotes blood vessel invasion and distant metastasis in triple-negative mammary tumors. *Cancer Research*.

[6] Dangi-Garimella S., Krantz S. B., Barron M. R. (2011). Three-dimensional collagen-I promotes gemcitabine resistance in pancreatic cancer through MT1-MMP-mediated expression of HMGA2. *Cancer Research*.

[7] Pahwa S., Stawikowski M. J., Fields G. B. (2014). Monitoring and inhibiting MT1-MMP during cancer initiation and progression. *Cancers*.

[8] Overall C. M., López-Otín C. (2002). Strategies for MMP inhibition in cancer: innovations for the post-trial era. *Nature Reviews Cancer*.

[9] Kaimal R., Aljumaily R., Tressel S. L., Agarwal A. (2013). Selective blockade of matrix metalloprotease-14 with a monoclonal antibody abrogates invasion, angiogenesis, and tumor growth in ovarian cancer. *Cancer Research*.

[10] Botkjaer K. A., Kwok H. F., Terp M. G. (2016). Development of a specific affinity-matured exosite inhibitor to MT1-MMP that efficiently inhibits tumor cell invasion *in vitro* and metastasis *in vivo*. *Oncotarget*.

[11] Devy L., Huang L., Naa L. (2009). Selective inhibition of matrix metalloproteinase-14 blocks tumor growth, invasion, and angiogenesis. *Cancer Research*.

[12] Ingvarsen S., Porse A., Erpicum C. (2013). Targeting a single function of the multifunctional matrix metalloproteinase MT1-MMP: impact on lymphangiogenesis. *Journal of Biological Chemistry*.

[13] Gálvez B. G., Matías-Román S., Albar J. P., Sánchez-Madrid F., Arroyo A. G. (2001). Membrane type 1-matrix metalloproteinase is activated during migration of human endothelial cells and modulates endothelial motility and matrix remodeling. *Journal of Biological Chemistry*.

[14] Udi Y., Grossman M., Solomonov I. (2015). Inhibition mechanism of membrane metalloprotease by an exosite-swiveling conformational antibody. *Structure*.

[15] Zhu L., Wang H., Wang L. (2011). High-affinity peptide against MT1-MMP for *in vivo* tumor imaging. *Journal of Controlled Release*.

[16] Kondo N., Temma T., Shimizu Y. (2015). Radioiodinated peptidic imaging probes for *in vivo* detection of membrane type-1 matrix metalloproteinase in cancers. *Biological & Pharmaceutical Bulletin*.

[17] Min K., Ji B., Zhao M. (2015). Development of a radiolabeled peptide-based probe targeting MT1-MMP for breast cancer detection. *PLoS One*.

[18] Temma T., Sano K., Kuge Y. (2009). Development of a radiolabeled probe for detecting membrane type‐1 matrix metalloproteinase on malignant tumors. *Biological & Pharmaceutical Bulletin*.

[19] de Lucas A. G., Schuhmacher A. J., Oteo M. (2016). Targeting MT1-MMP as an ImmunoPET-based strategy for imaging gliomas. *PLoS One*.

[20] Perk L. R., Vosjan M. J. W. D., Visser G. W. M. (2010). p-Isothiocyanatobenzyl-desferrioxamine: a new bifunctional chelate for facile radiolabeling of monoclonal antibodies with zirconium-89 for immuno-PET imaging. *European Journal of Nuclear Medicine and Molecular Imaging*.

[21] Vosjan M. J. W. D., Perk L. R., Visser G. W. M. (2010). Conjugation and radiolabeling of monoclonal antibodies with zirconium-89 for PET imaging using the bifunctional chelate p-isothiocyanatobenzyl-desferrioxamine. *Nature Protocols*.

[22] Romero E., Morcillo M. A. (2017). Inorganic oxides with potential application in the preparation of a ^68^Ge/^68^Ga generator system. *Applied Radiation and Isotopes*.

[23] Romero E., Martinez A., Oteo M. (2016). Preparation of 68Ga-labelled DOTA-peptides using a manual labelling approach for small-animal PET imaging. *Applied Radiation and Isotopes*.

[24] Rubio-Viqueira B., Jimeno A., Cusatis G. (2006). An in vivo platform for translational drug development in pancreatic cancer. *Clinical Cancer Research*.

[25] Hidalgo M., Bruckheimer E., Rajeshkumar N. V. (2011). A pilot clinical study of treatment guided by personalized tumorgrafts in patients with advanced cancer. *Molecular Cancer Therapeutics*.

[26] Yushkevich P. A., Piven J., Hazlett H. C. (2006). User-guided 3D active contour segmentation of anatomical structures: significantly improved efficiency and reliability. *Neuroimage*.

[27] Ellenrieder V., Alber B., Lacher U. (2000). Role of MT-MMPs and MMP-2 in pancreatic cancer progression. *International Journal of Cancer*.

[28] Zhang L., Bhatnagar S., Deschenes E., Thurber G. M. (2016). Mechanistic and quantitative insight into cell surface targeted molecular imaging agent design. *Scientific Reports*.

[29] Gengenbacher N., Shingal M., Augustin H. G. (2017). Preclinical mouse solid tumour models: status quo, challenges and perspectives. *Nature Reviews Cancer*.

[30] Jensen M. M., Jorgensen J. T., Binderup T. (2008). Tumor volume in subcutaneous mouse xenografts measured by microCT is more accurate and reproducible than determined by ^18^F-FDG-microPET or external caliper. *BMC Medical Imaging*.

[31] Fragogeorgi E. A., Zikos C., Gourni E. (2009). Spacer site modifications for the improvement of the *in vitro* and *in vivo* binding properties of (99m)Tc-N(3)S-X-bombesin2-14 derivatives. *Bioconjugate Chemistry*.

[32] Buchsbaum D. J., Langmuir V. K., Wessels B. W. (1993). Experimental radioimmunotherapy. *Medical Physics*.

[33] Jalilian A. R., Osso J. A. (2017). Production, applications and status of zirconium-89 immunoPET agents. *Journal of Radioanalytical and Nuclear Chemistry*.

[34] Heskamp S., Raavé R., Boerman O. (2017). ^89^Zr-immuno-positron emission tomography in oncology: state-of-the-art ^89^Zr radiochemistry. *Bioconjugate Chemistry*.

[35] McDonald D. M., Choyke P. L. (2003). Imaging of angiogenesis: from microscope to clinic. *Nature Medicine*.

[36] Deri M. A., Zeglis B. M., Francesconi L. C. (2013). PET imaging with ^89^Zr: from radiochemistry to the clinic. *Nuclear Medicine and Biology*.

[37] Chakravarty R., Goel S., Cai W. (2014). Nanobody: the “magic bullet” for molecular imaging?. *Theranostics*.

[38] Krasniki A., D´Huyvetter M., Devooggdt N. (2018). Same-day imaging using small proteins: clinical experience and translational prospects in oncology. *Journal of Nuclear Medicine*.

[39] Imamura T., Ohshio G., Mise M. (1998). Expression of membrane-type matrix metalloproteinase-1 in human pancreatic adenocarcinomas. *Journal of Cancer Research and Clinical Oncology*.

[40] Bramhall S. R., Neoptolemos J. P., Stamp G. W. (1997). Imbalance of expression of matrix metalloproteinases (MMPs) and tissue inhibitors of the matrix metalloproteinases (TIMPs) in human pancreatic carcinoma. *Journal of Pathology*.

[41] Ottaviano A. J., Sun L., Ananthanarayanan V. (2006). Extracellular matrix-mediated membrane-type 1 matrix metalloprotease expression in pancreatic ductal cells is regulated by transforming growth factor-ß1. *Cancer Research*.

[42] Ying H., Dey P., Yao W. (2016). Genetics and biology of pancreatic ductal adenocarcinoma. *Genes & Development*.

[43] Siegel R. L., Miller K. D., Jemal A. (2017). Cancer statistics. *CA: A Cancer Journal for Clinicians*.

[44] Garrido-Laguna I., Hidalgo M. (2015). Pancreatic cancer: from state-of-the-art treatments to promising novel therapies. *Nature Reviews Clinical Oncology*.

[45] Liang C., Shi S., Meng Q. (2017). Complex roles of the stroma in the intrinsic resistance to gemcitabine in pancreatic cancer: where we are and where we are going. *Experimental & Molecular Medicine*.

